# Managing Clutter in a High Pulse Rate Echolocation System

**DOI:** 10.3389/fnins.2018.00177

**Published:** 2018-03-21

**Authors:** Jacob Isbell, Timothy K. Horiuchi

**Affiliations:** ^1^Electrical and Computer Engineering Department, University of Maryland, College Park, College Park, MD, United States; ^2^Institute for Systems Research, University of Maryland, College Park, College Park, MD, United States; ^3^Neuroscience and Cognitive Science Department, University of Maryland, College Park, College Park, MD, United States

**Keywords:** echolocation, sonar, bats, clutter, interpulse interval, pulse-echo ambiguity

## Abstract

The use of echolocation for navigating in dense, cluttered environments is a challenge due to the need for rapid sampling of *nearby* objects in the face of delayed echoes from *distant* objects. In the wild, echolocating bats frequently encounter this situation when leaving the roost or while hunting. If long-delay echoes from a distant object are received after the next pulse is sent out, these “aliased” echoes appear as close-range phantom objects. Little is known about how bats cope with these situations. In this work, we demonstrate a novel strategy to manage aliasing in cases where a single target is actively being tracked at close range. This paper presents three reactive strategies for a high pulse-rate sonar system to combat aliased echoes: (1) changing the interpulse interval to move the aliased echoes away in time from the tracked target, (2) changing positions to create a geometry without aliasing, and (3) a phase-based, transmission beam-shaping strategy to illuminate the target and not the aliasing object.

## Introduction

Bat echolocation is the unusual ability by bats to emit an ultrasonic sound pulse and measure the time until echoes begin to arrive (for estimating range) combined with the more general ability of mammals to determine the direction of sound. The ultrasonic frequencies used by bats are difficult to detect by most animals and have short wavelengths (~ 3–17 mm) that produce detectable echoes from small insects. To localize the direction of echoes, bats (e.g., the big brown bat) have been shown to rely primarily on the use of interaural level differences produced by the head and pinnae, a common strategy for small mammals (Grothe et al., 2010). The use of ultrasonic frequencies and a small head size, strongly limit the use of phase-locking, and interaural-timing cues for localization. To estimate range, the bat measures the time-of-flight of the echo from an emitted sound. From an auditory processing point of view, echolocation is unique in that the sound being analyzed is *generated* by the bat and is therefore both known and under the control of the bat. It is well known that bats change both the properties of the echolocation pulse and the timing of pulses in response to their environment (Petrites et al., 2009; Hiryu et al., 2010; Bates et al., 2011), but seldom has this dynamic behavior been adopted in artificial sonar systems.

A typical operational assumption in echolocation is that all of the sounds following an emitted pulse are echoes from the *most recent* outgoing pulse. The duration of perceptible echoes resulting from a given pulse depends on the properties of the outgoing pulse (such as the amplitude, spectrum, and duration) as well as the properties of the environment (such as the distance, size, shape, orientation, and overall configuration of objects). A common-sense rule is that the next pulse should not be emitted until *all* perceptible echoes from the previous pulse have died out. In the majority of situations, bats appear to avoid this pulse-echo ambiguity, or “aliasing.” Studies of big brown bats navigating in extremely cluttered environments, however, show cases where bats appear to tolerate such aliasing to sample the environment at a high-rate (Petrites et al., 2009; Schmidt et al., 2011).

In close-quarters maneuvering, a high sampling rate is desirable when the angle to nearby objects is changing rapidly. Little is known about what bats do when a high pulse rate is needed to maneuver near objects in an environment that produces long-delay echoes, a situation that produces echo aliasing. Big brown bats have been shown to alternate between pulsing rapidly and pulsing slowly. Pulsing rapidly gives a clearer picture for close ranges while pulsing slowly gives a clearer picture for long ranges (Petrites et al., 2009). Another possible strategy might be to reduce the intensity of the call or reduce the low-frequency components of the chirp to reduce the distance over which the perceptible acoustic pulse travels. Bats have also been observed to change the spectral content of consecutive pulses, largely by shifting the entire pulse up or down in frequency. The spectral signature of the returned echoes can then be used to assign them to a specific pulse (Hiryu et al., 2010). This technique has also been used in radar (Gokturk et al., 2004; Skolnik, 2008) to increase the effective sampling rate. Another technique utilized by radar systems is to transmit multiple pulses in a short temporal pattern (or “code”). Different codes can then be used to identify different pulses (Skolnik, 2008; Matsuta et al., 2013). When the task is to track a specific target object (e.g., an obstacle the bat is maneuvering around), an attentional mechanism can be used to ignore the background and any aliasing that may be occurring. This approach works well until an “aliased” echo arrives at or near the time of the tracked echo. Three strategies for avoiding aliased echoes are presented: (1) a dynamic pulse-timing strategy that would allow a bat to “push” aliased echoes away from the attended window in time (adaptive delay), (2) changing the sonar “viewing angle” to the target to change the background (movement), and (3) using temporal phasing of two transducers during transmission to create an interference pattern in the sonar beam (with peaks and valleys) that can be used to isolate the target object (beam shaping).

## Materials and methods

### Hardware

**Figure 1:**
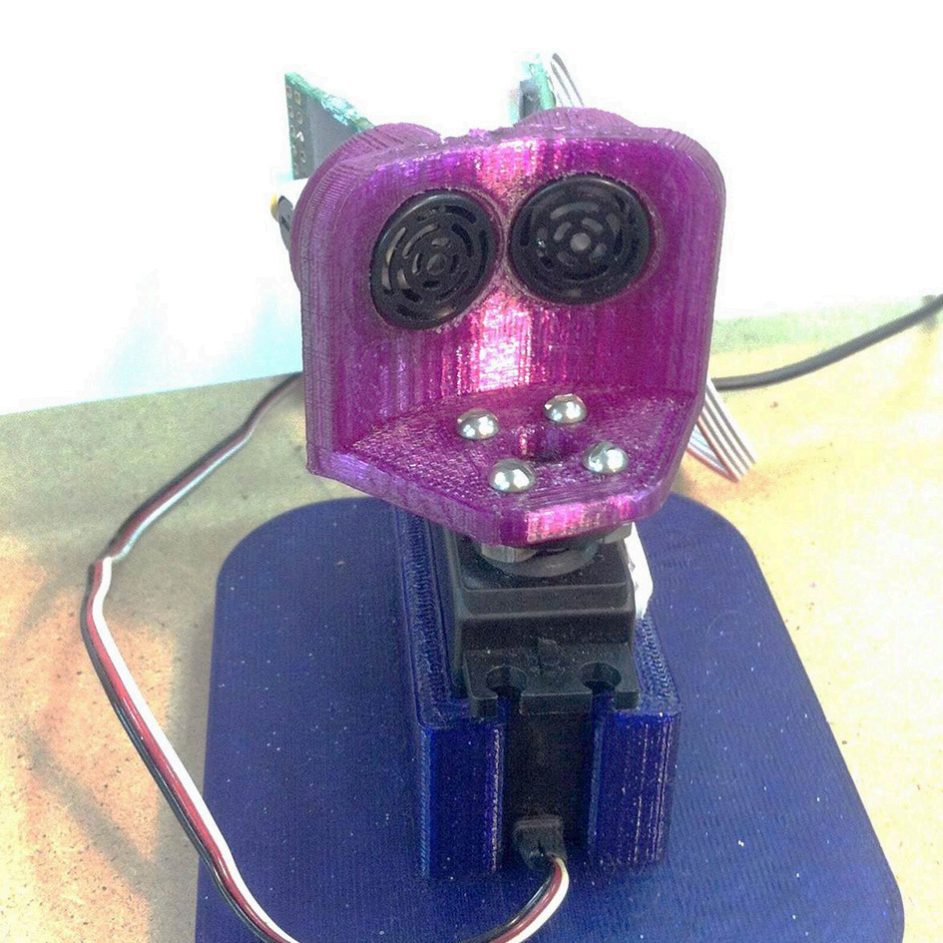
The A two transducer sonar head mounted on a hobby servomechanism that was used for the experiments in this paper. The sonar modules are a custom 40 kHz system modified from a high-power Maxbotix sonar.

The sonar system used in the work presented here consists of two custom modified MaxBotix® sonar transducers (shown in Figure 1), a custom PIC® 18F2620^1^ (Microchip Technologies Inc.) (MaxBotix Inc., 2016) microcontroller-based sonar controller board, a Futaba S148 hobby servo, and a computer interface to both record and display echo signals and control the servo to orient the sonar. The transducers act as both a speaker and a microphone. They resonate specifically at 40 kHz and will only detect signals near this frequency. The custom sonar boards report a logarithmically-compressed envelope signal as an analog voltage. This allows the output to report the very wide dynamic range of amplitudes that occurs with sonar without saturating. The transducers are placed in a 3-D printed mount on the servo motor. In this demonstration system, the transducers transmit and receive over a cone of about ±30°, so the transducers are held facing 30° apart to ensure sufficient overlap and coverage of the area in front of the transducers for binaural localization. The ultrasonic pulse trigger-timing and analog-to-digital (A/D) conversion is done by the microcontroller. The majority of the data processing is performed on the microcontroller to ensure a quick response. Echo data is transferred via serial interface to a PC and the PC controls the servo motor via a USB-interfaced servo control board (Pololu, 2017).

### The tracking cycle

**Figure 2:**
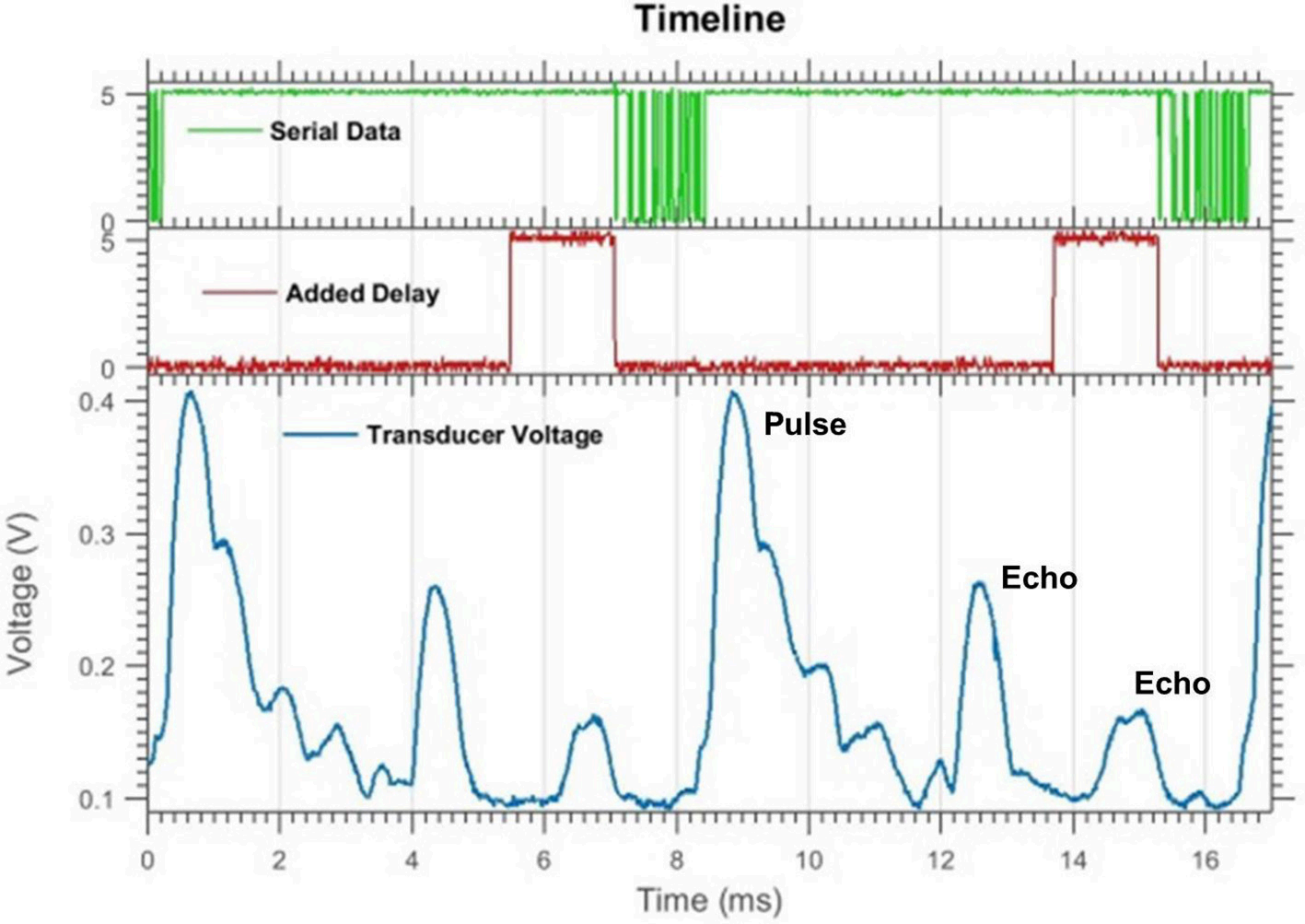
An oscilloscope readout of two pulse-echo cycles (without aliasing) showing the transducer envelope voltage (**bottom**), serial data transfer (**top**), and added delay (**middle**). The added delay flag is set high when the delay is occurring. Objects can be seen as distinct peaks in the transducer voltage trace. Pulsing and sampling the transducer takes 5 ms, then there is a 1.5 ms delay and 1.5 ms of serial data transfer. The whole cycle takes about 9 ms.

The sonar system executes four repeated steps: pulsing, sampling, processing, and communicating. As part of the cycle, there is an added delay interval that is used to reject aliased echoes (discussed in section Adaptive Delay). A few of these steps are shown in Figure 2 for two cycles. In these examples, a short duration ultrasonic command pulse (~0.25 ms) is used, however, due to the resonant quality of the transducer, the duration of the acoustic pulse is extended. Following the pulse, the transducer continues to ring for several milliseconds. Although echoes can be detected during this ringing period, their amplitudes are difficult to estimate, so a short 2 ms delay (i.e., dead-zone) is incorporated before sampling begins. The log-compressed envelope voltage is sampled every eighth of a millisecond. Object detection begins when the temporal derivative of the envelope exceeds a threshold of approximately 3.4 dB over an eighth of a millisecond. Once the peak of the envelope has been reached, the object range is determined by the time since emission and the direction is estimated using the amplitudes on the two transducers.

At low pulse rates, the echoes are monitored for a period of time associated with the maximum range of the sonar and an extra delay would be added after transmitting the recorded data. In the case of fast pulsing where a target is being tracked, once the target echo is received, a short data burst is transmitted and the next cycle is initiated. After detecting the target echo, the tracking window (in time) is updated and the intensities of both transducers are compared to rotate the servo motor to center the target echo. At this time, temporal windows before and after the target echo are monitored to detect if other echoes are about to overlap with the target echo. This information is used to initiate the various reactive strategies to avoid interference with target tracking (described in sections Adaptive Delay, Movement, and Beam Shaping).

### Target tracking

There are occasions when the echo from the target disappears completely due to interference or occlusion by an object in the foreground. The tracker continues to search for the target at the same range for up to three cycles after the object disappears. If the object does not reappear, it will begin looking for a new target at a pre-specified acquisition range.

For the purposes of this study, the tracker is programmed to initially find the target at a single, pre-specified range (about 33 cm) and then follow it in range and in the horizontal plane by turning the sensor head to center the object. Centering is accomplished by rotating the sensor head until the detected amplitudes of the target in the two transducers are approximately equal. Only horizontal angles are considered. Since the echo amplitude is logarithmically compressed, the difference between left and right outputs corresponds to a ratio of the two received amplitudes. This ratio (invariant to echo amplitude) can be mapped to a specific angle. This mapping is defined by the spatial sensitivity and placement angles of the receivers and is found empirically. The ratio is monotonic and allows for reasonable angle measurements over a range of ±30°. Outside of this region, only one transducer will produce a significant response, allowing only a coarse approximation of direction. The response of our system at various angles is discussed in the Beam Shaping section.

The range of objects is determined by the time when the echo is received (i.e., time-of-flight). In practice, this is a very stable measurement that is minimally affected by noise. The echo amplitude, however, is very sensitive to factors such as the shape and orientation of the target, interfering reflections and echoes, and positioning of the transducers. At high repetition rates, a reverberant room can become filled with sound, introducing significant background interference. To avoid wild oscillations in the servo motor pointing, the system is restricted to moving a maximum step of 5° between echoes.

Once an object is found at the pre-specified acquisition range, it is labeled as the target and tracked. In the next pulse cycle, the sonar will expect to receive an echo within 6.3 cm of the previous target range. By restricting the temporal size of the tracking box, all echoes other than the target are ignored allowing the system to track a single object in the midst of other objects. Analog-to-digital sampling is performed with a period of an eighth of a millisecond and thus the range resolution is 2.1 cm/sample.

### Aliasing and clutter

**Figure 3:**
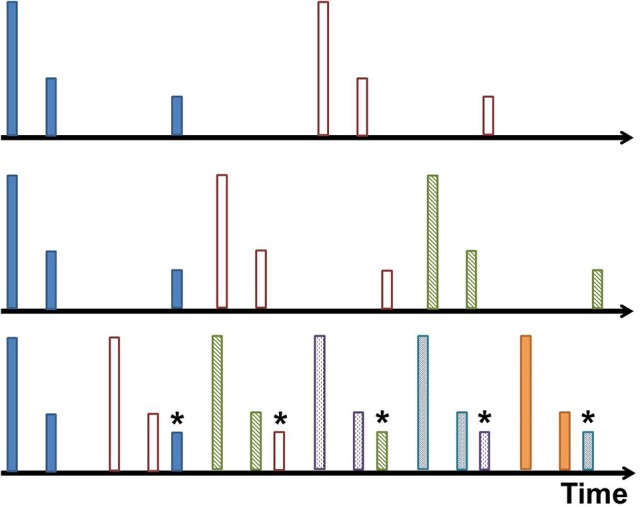
Aliasing visualized. In this cartoon example, each timeline has pulses (represented by tall lines) and received echoes (represented by shorter lines). Each pulse and its echoes are given a unique color. From top to bottom, the interpulse interval decreases until a new pulse occurs before all echoes from the previous pulse are received, shown in the bottom timeline. The echo is misinterpreted as a closer object associated with the latest pulse. This is the aliased echo, and is labeled with an asterisk.

**Figure 4:**
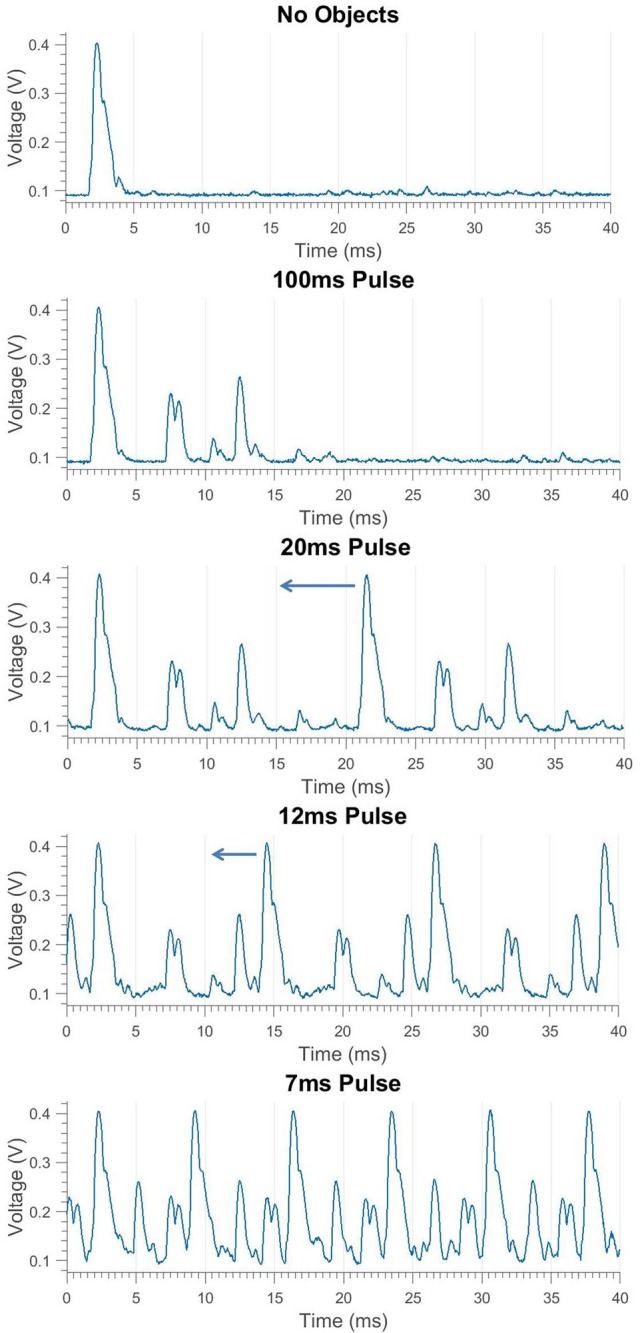
Transducer envelope of pulses and echoes at different repetition rates demonstrating aliasing in the bottom graph. The outgoing pulse peaks at 0.4 V, overlapping echoes from two closely-spaced PVC pipes are seen peaking at 0.2 V, and a single loud echo made by a square poster board is seen peaking at 0.25 V. The interpulse interval is decreased in each graph until a new pulse occurs before all echoes from the previous pulse are received, causing an aliasing condition where the poster board incorrectly appears at short range.

In the rapid pulse mode, the maximum detection range for the sonar system is limited by the interpulse interval. If an object has an echo time that is greater than one pulse period, it is detected by the system in the next pulse cycle. It is then perceived as having an echo time that is one pulse period less than it actually is. Since this distortion is caused by sampling related to each pulse, we call it aliasing. This is demonstrated in Figures 3, 4. While the perceived direction of this “phantom” object is unchanged, the range is wildly incorrect and may even overlap the echo from the tracked target. The techniques presented in this paper aim to keep the range and angle measurements of the target clean. This can be done by keeping other echoes far enough away (in time) to not overlap the target echo (~ 0.5 ms). If that is not possible, the goal is to reduce the amplitude of the obstructing echo as much as possible.

Two strategies specific to problem of aliased echoes overlapping the target echo are presented: First, by using an **adaptive delay**, the interpulse interval can be manipulated to change the relative time of the aliased echo. This changes the perceived range of the alias to prevent it from overlapping with the target. Second, the sonar system can use **movement** to prevent objects in the background from falling in the main path of the sonar beam. This reduces the magnitude of clutter echoes.

These strategies may not always work, particularly if the aliased object is close in range to the target and the sonar beam is too wide for the movement strategy to avoid illuminating the aliasing object. In this case, **beam forming** of the transmitted pulse by firing both transducers in a phased manner can be used to increase the amplitude of the target echo and decrease the amplitude of the aliased object echo. This can also be effective in non-aliasing situations where a distractor object at the same range (but different angle) is causing interference.

### Adaptive delay

**Figure 5:**
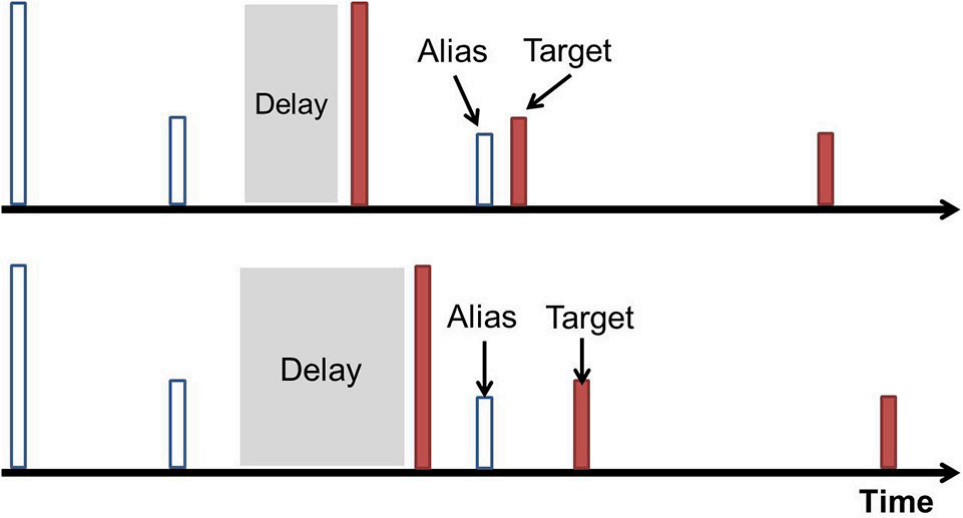
Manipulating the received time of an aliased echo. The tall line represents the pulse and the short lines represent the echoes. The echoes associated with a given pulse are the same color. The top timeline shows an alias (white) that is close to interfering with the first dark echo, the target. The introduced delay is increased (in the bottom timeline) to shift this aliased echo away from the target in time. Similarly, an alias on the other side of the target can also be shifted away by decreasing the delay (not shown).

The range at which the aliased echo appears is dependent on the time between sending pulses. To control this, a variable delay period is inserted before sending the next pulse. Increasing this delay shortens the aliased echo time, making it appear to move closer to the sonar. Decreasing the delay increases the aliased echo time, making it appear to move away from the sonar (an example is shown in Figure 5).

The alias rejection system introduces a delay interval with a maximum of 3 ms into the timeline. The interval length is changed in eighth millisecond increments based on where the aliased echo appears relative to the tracked target. If an aliased echo is within 5 range samples, or 10.7 cm, of the target echo, the delay interval will be changed to repel the aliased echo. For an aliased echo that appears closer than the target echo (i.e., in between the target and the sonar system), we increase the delay to move the aliased echo away from the target echo; an aliased echo further away than the target echo decreases the delay. If the delay reaches its maximum amount or if it is decreased to zero, the delay value is reset to 1.5 ms (half of its maximum value). This will cause an aliased echo to jump to the other side of the target echo, being shifted by 12 range samples. If there is an aliased echo detected on both sides of the target, the delay is shifted by a large amount, equivalent to 11 range samples, in an attempt to clear both aliased echoes away from the target echo. This process is summarized below.

**If** alias in front

     **Increase** delay

**If** alias in back

     **Decrease** delay

**If** alias in front **and** alias in back

     Large delay **shift**

**If** delay is minimum **or** delay is maximum

     **Reset** delay

#### Checking for real objects

While we have assumed a relatively isolated target object to track, a real second object in close proximity to the target cannot be “rejected.” In this case, the alias rejection system would continuously shift the delay, resulting in oscillations of the delay shifting and resetting when the delay interval reaches its limits. To prevent these oscillations, additional code is used to recognize authentic (i.e., non-aliased) echoes.

The most notable difference between an authentic echo and an aliased echo is their reaction to a large shift in the interpulse interval, a delay jump. An alias will be moved a significant amount, while an authentic echo will not be moved at all. Although a real object can still move noticeably, at low speeds (<3 m/s) it will not jump more the one range sample at a time.

The alias rejection system makes large delay shifts in three different scenarios: when the delay interval reaches its maximum, its minimum, and when two aliases sandwich the target (one on either side). The system uses these events as triggers to look for an authentic echo that remains in the same location. This is especially appropriate since an authentic echo triggers an oscillation that causes the delay to jump when the interval reaches a maximum or minimum. If an object doesn't move after a delay jump, it is recognized as an authentic echo and will not activate the alias rejection system. This is similar to a technique used in radar where a map of stationary clutter is memorized and removed (Skolnik, 2008).

### Movement

An alternative method to avoid sonar aliasing is to reposition the sonar beam such that objects in the background do not generate echoes. The effectiveness of this technique will depend on using a relatively narrow transmission beam. Depending on the species of bat, transmission beam widths can range from 22 to 90° (Jakobsen and Surlykke, 2010; Nachtigall and Moore, 2012; Matsuta et al., 2013). The sonar beam width used in this study is approximately 30°.

When the sonar moves around, different sides of objects are exposed to the sonar. In general, this will complicate a decision to change the sensing angle, since the acoustic properties of an object can change greatly from different perspectives. To demonstrate this, two different objects were used as the aliasing object in two different trials: a large 46 cm (1.5 ft) diameter cardboard tube and a 30 cm wide, open cardboard box. The sonar was moved around a target object to continue aiming the beam at the target at the same range, but resulting in different backgrounds (Figure 9). As the sonar moves, the transmission beam is moved away from the aliasing object and the magnitude of its echo decreases. Theta is the angle of rotation the sonar system has made around the target relative to its starting location. For this study, only one transducer was used.

### Beam shaping

A third strategy for reducing the effect of aliasing and clutter objects is to shape the acoustic beam so that only the target object is ensonified. With the two-transducer system used in the study, this is performed by transmitting with both transducers to create an interference pattern that has peaks and nulls that can be used to reduce interference. Plots of the beam shape are shown for a single transducer, the two transducers firing synchronously, and the two transducers firing out-of-phase (Figure 11). The synchronous in-phase firing pattern has a loud frontal lobe that is relatively narrow with weaker lobes on either side. The −6 db width of the front lobe is 19° (compared to 62° of a single transducer alone). The stronger, narrower central lobe would allow more precise ensonification of a target while reducing echoes from other directions. It is important to note that the patterns presented here represent the transmitted beam only. The sonar hardware presented here does not allow phased detection; although that is an additional capability in other systems that would further improve selectivity.

Figure 12 shows an example of how using this firing pattern can affect an echo trace. There are two objects in the field of view, both PVC pipes of equal diameter. Using only a single transducer without phasing, the clutter echo impinges on the target echo and disrupts the information conveyed. Using two transducers fired synchronously, the cluttered object has a significantly reduced magnitude and the target will not be as affected by the clutter. This example, however, represents a best-case scenario where the clutter object falls in the low trough of the firing pattern. Utilizing this system in arbitrary object configurations is not trivial. Since angle estimation is very noisy in this sonar system, predicting the effects of beam shaping can be error-prone and not guaranteed to be beneficial.

The experimental configuration used is shown in Figure 13. With the target centered in the sonar view, the clutter object is moved to different angles relative to the center. Both objects are 5 cm diameter PVC pipes at a range of 122 cm (4 ft).

## Results

### Adaptive delay

**Figure 6:**
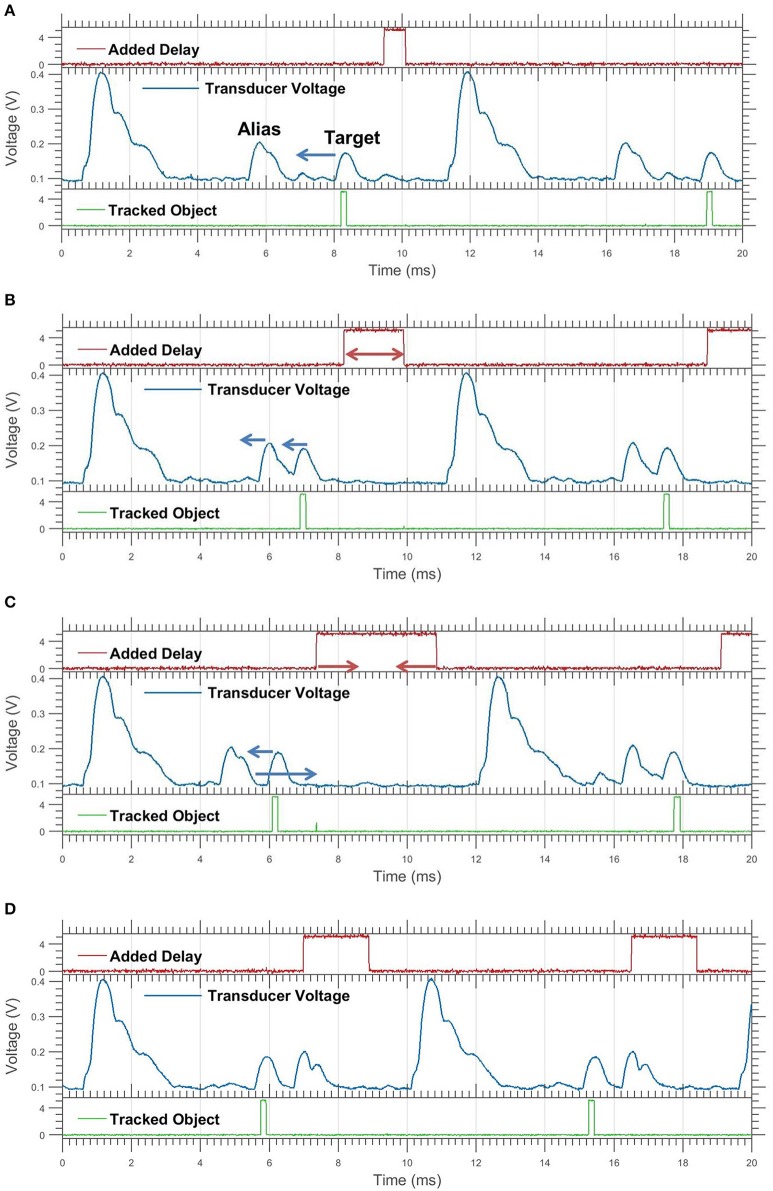
Oscilloscope showing transducer voltage, delay, and tracking for an *approaching target*. The added delay bit is high when the delay is occurring. The tracking bit is high when receiving the echo of the object being tracked. These graphs are a sequence of events in real time **(A–D)**. Only two significant objects are present, the target marked by the tracking bit, and the aliased echo. The only object moved was the target; the apparent movement of the alias is due to the delay change. The arrows show movement change for next frame. **(A,B)** The target moves forward, toward the alias. **(B,C)** The target continues forward, the alias is pushed forward by the increasing delay. The delay buffer becomes maximized. **(C,D)** The delay buffer jumps down after reaching its maximum. This causes the alias to “jump” behind the target.

**Figure 7:**
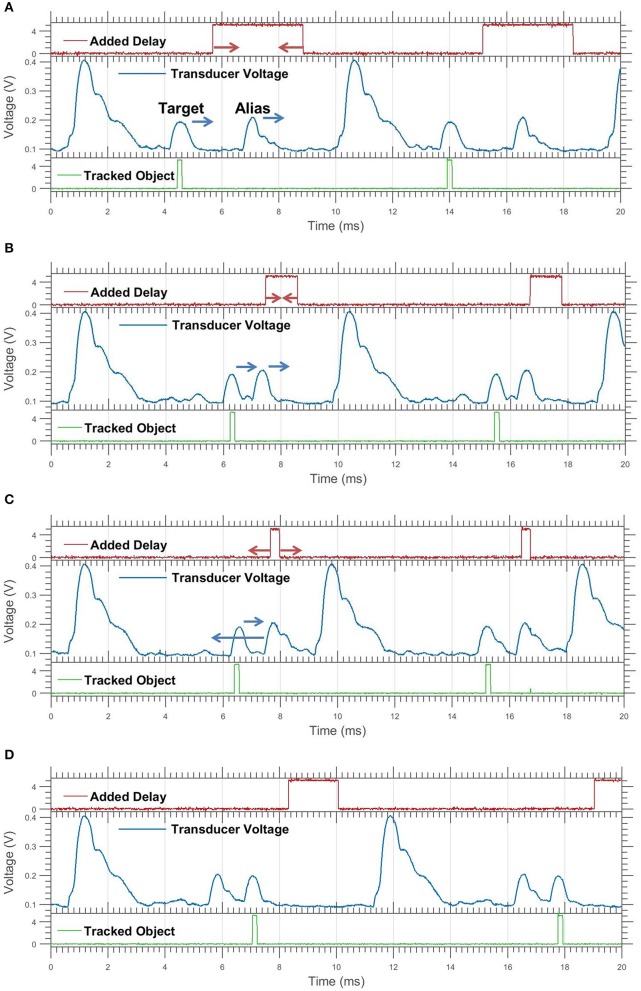
Continuation of Figure 6 for a *retreating target*. **(A,B)** The target moves back; the alias is pushed back by the decreasing delay. **(B,C)** Both echoes continue backwards, the delay buffer reaches its minimum value. **(C,D)** The delay buffer jumps upwards, causing the alias to jump forwards in front of the target.

**Figure 8:**
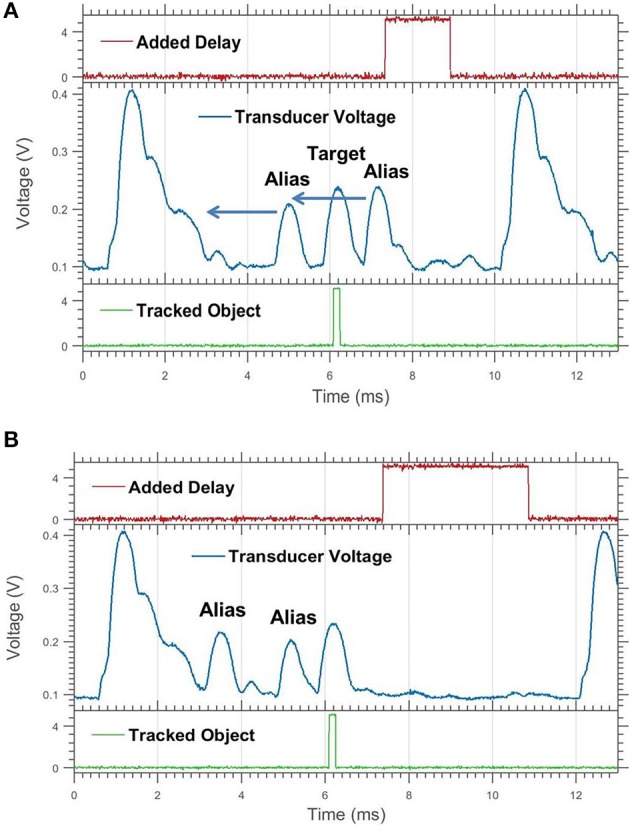
A target being sandwiched by aliased echoes. **(A)** Here the alias in front of the stationary target approaches the target and triggers a delay jump. **(B)** This clears both aliases away from the target, drawing them both forward of the target.

Figures 6–8 show the system in action, presenting consecutive graphs in time that demonstrate system functionality. The three figures represent the three different cases of aliases: aliases moving toward the target from the front, aliases moving toward the target from the back, and two aliases sandwiching the target. In each case it is assumed that the target starts clear and unobscured. The adaptive delay prevents the target from becoming obscured in all the cases.

**Figure 9:**
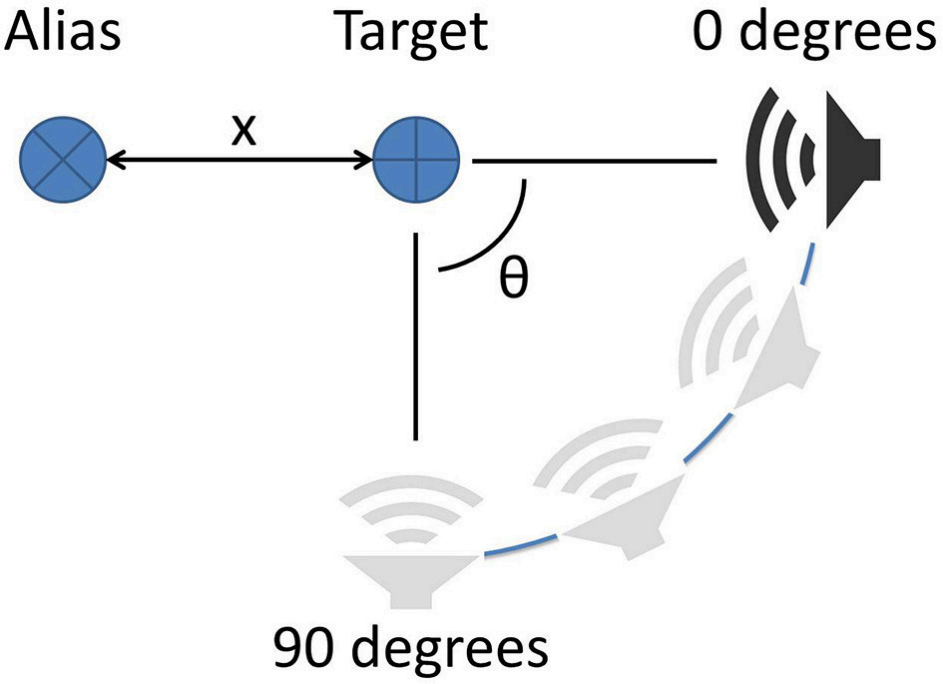
Alias rejection via movement. The sonar system (speaker) is kept a constant distance from the target. The alias is located at a distance *x* from the target. The sonar is rotated around the target by angle θ to shift the view of the system.

### Movement

**Figure 10:**
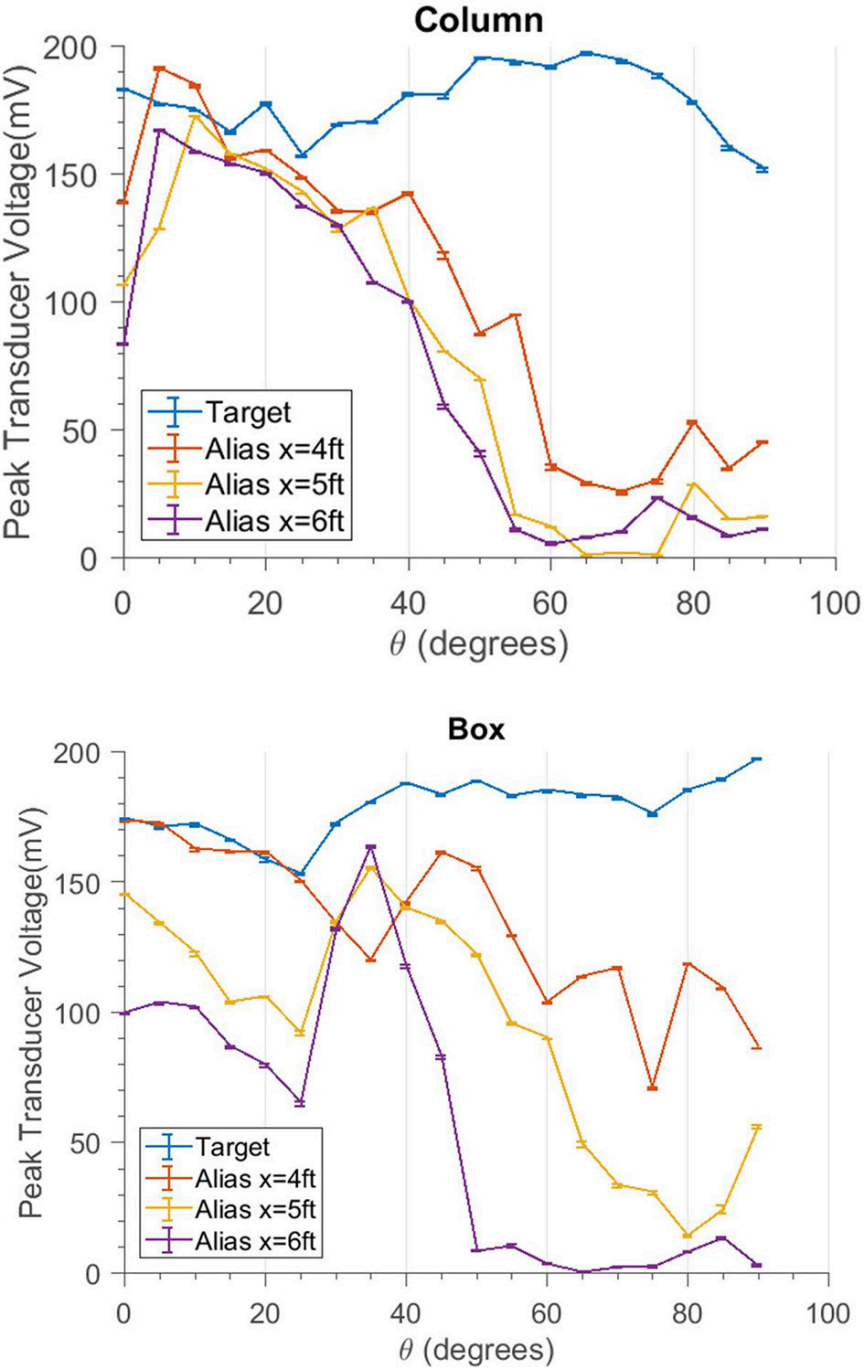
Traces showing the echo response of the target and the alias at different angles. The target trace (blue) gives a baseline for comparison. The “Alias” traces reduce in amplitude as the angle increases. For larger distances *x* the amplitude decreases even more.

Figure 10 shows the results of the movement study with the aliasing object at 4, 5, and 6 ft (labeled x) away from the target. At the same angle, larger x values push the aliasing object farther away from the center of the beam and cause a larger reduction in magnitude. For the column, at a 60° angle of rotation, the aliased echo amplitude had been reduced to below 19% of the target echo amplitude for all distances of x. For the box at the same angle, the amplitude was only reduced to 57% of the target echo amplitude in the worst case (*x* = 4 ft). This highlights the role of the object geometry. It should be noted that the measured amplitudes are logarithmically-compressed acoustic amplitudes and the actual percentage change seen will vary with signal level.

**Figure 11:**
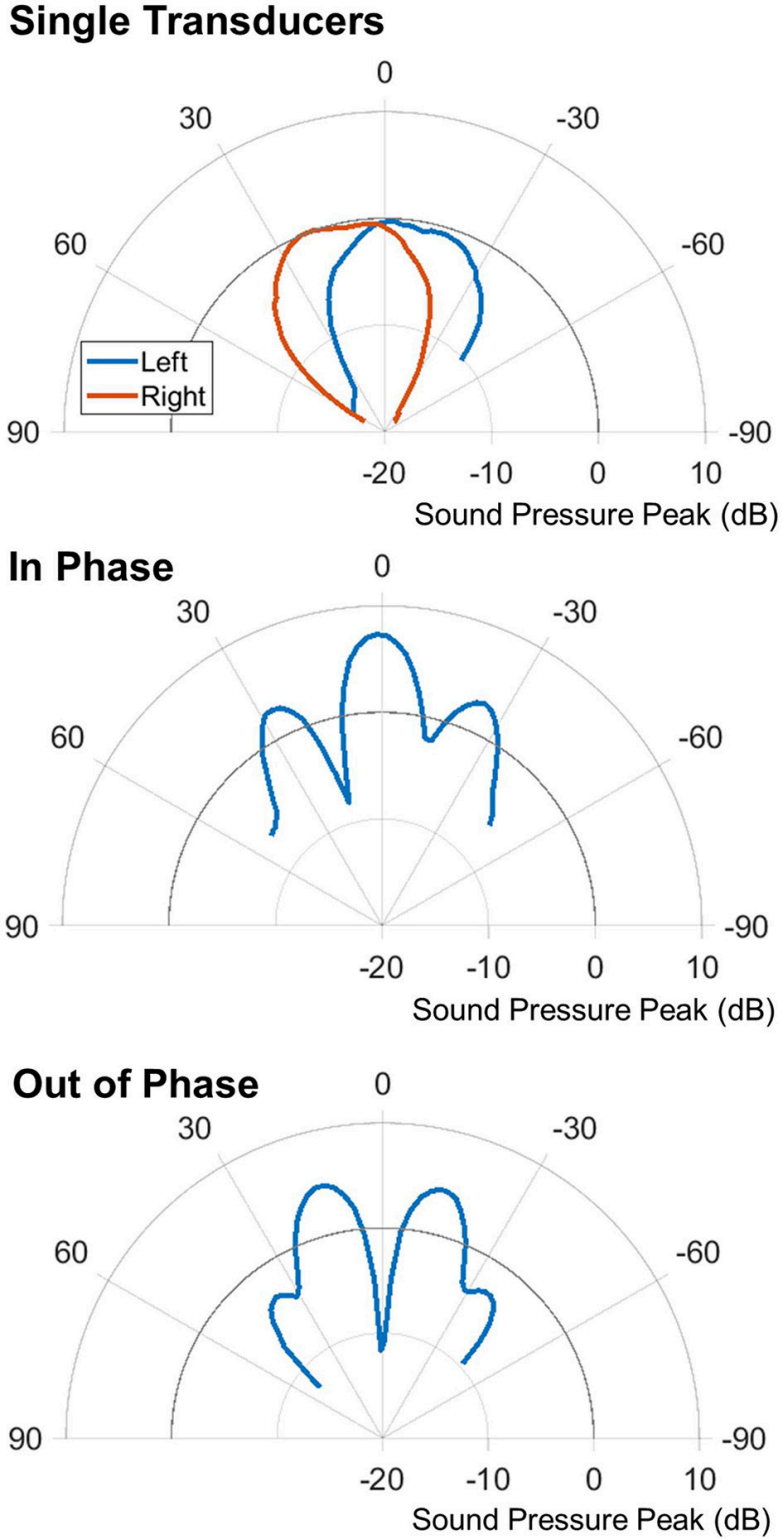
Polar plots of the different firing patterns. Top shows single transducer pulses from the left and right transducers. Middle shows the synchronous in-phase firing pattern. Bottom shows the synchronous out-of-phase firing pattern.

**Figure 12:**
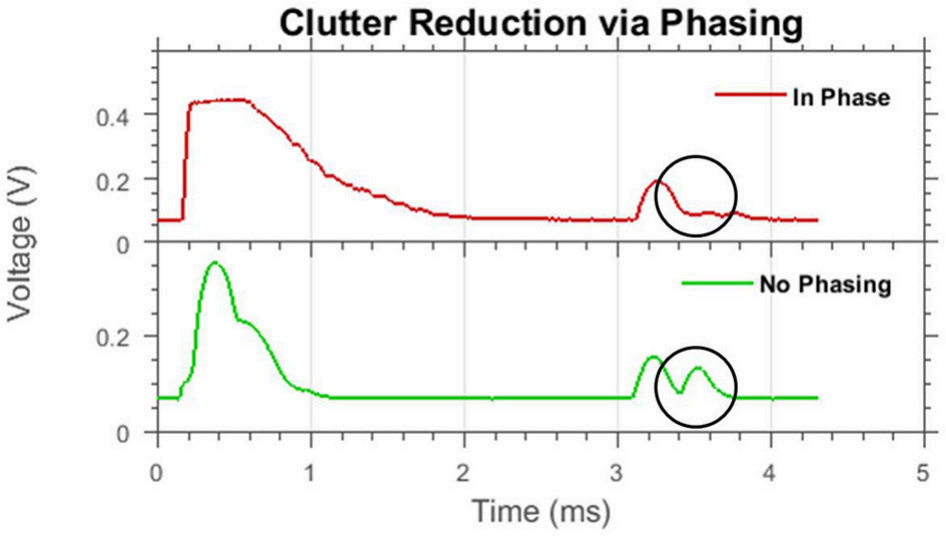
A best-case example of clutter reduction using beam shaping. Shown are two echo traces from the same scene with different beam shapes. Two objects are present, the first echo is the target object (~3.2 ms); the second echo is from the clutter object (~3.5 ms) which is circled. When in-phase firing is used, the clutter echo is greatly reduced in amplitude.

**Figure 13:**
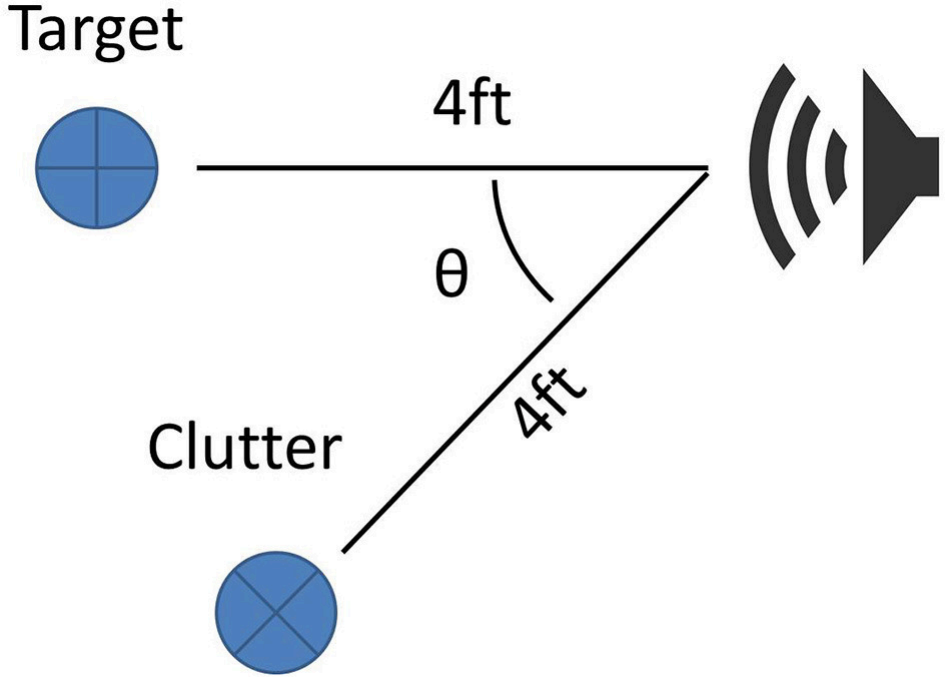
Clutter rejection using beam shaping. The sonar system faces a target that is 4 ft away. The clutter object is also 4 ft from the sonar but is rotated around the sonar system, changing its angle in the view of the sonar.

### Beam shaping

**Figure 14:**
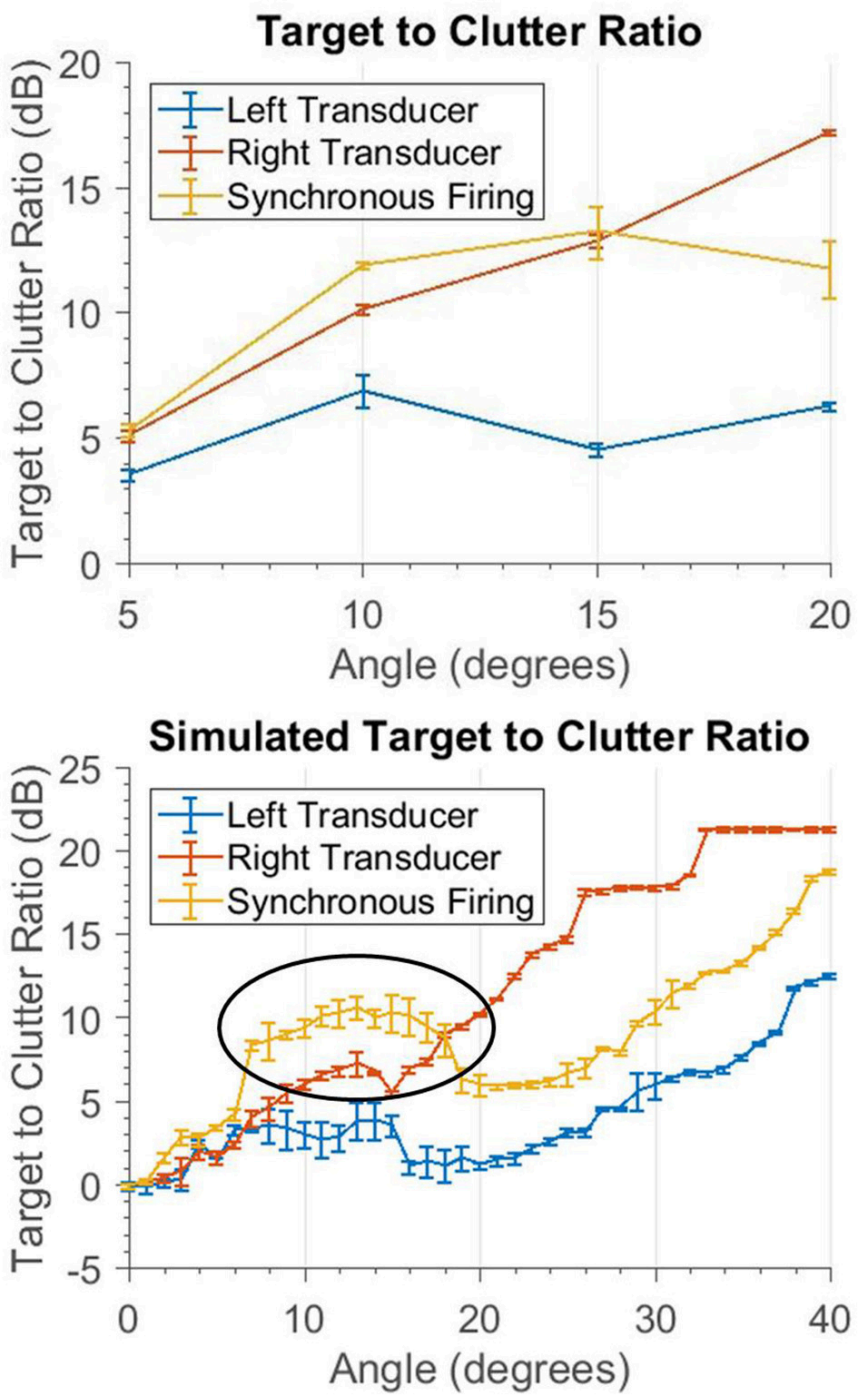
These graphs show how a clutter object appears at different angles. The target and clutter objects are at the same range. Only the angle to the clutter object is changed. The top graph shows the ratio of the target and clutter amplitude. The bottom shows simulated data, where only one object was scanned across all of the angles. The ratio was computed using the echo at angle 0 (i.e., the target) and the other angles (i.e., the clutter object). The circled area shows that for angles less than 18° the synchronous firing has better clutter rejection.

For the beam shaping study, the results are shown in Figure 14. The target to clutter (amplitude) ratio is used to normalize the data, which accounts for the difference in magnitude of the different firing patterns. The simulated data was created using the echoes from one real PVC pole recorded across all of the angles. The center measurement is used as the target amplitude; all other angles are treated as clutter amplitudes. The target to clutter ratio is calculated between the center and all other angles.

The synchronous firing pattern has a higher target to clutter ratio than the left or right transducers alone. This only occurs for angles less than 18°. This is due to the side lobes of the interference pattern; once the clutter starts to enter these lobes it is no longer sufficiently rejected and a single transducer will yield a better target to clutter ratio. In between 6 and 18°, where the most benefit is seen, there is a 3.39 dB average increase in the signal to clutter ratio with the synchronous firing pattern compared to the next best single transducer.

## Discussion

### Adaptive delay

The adaptive delay system for alias rejection tackles a problem that most engineered sonar systems avoid at the cost of a lower sampling frequency. When overlapping echolocation cycles are unavoidable, some form of pulse labeling is most commonly used (Uppala and Sahr, 1996; Gokturk et al., 2004; Skolnik, 2008; Hiryu et al., 2010; Matsuta et al., 2013). These techniques remove the issue of pulse-echo ambiguity since every pulse has its own unique characteristic. The approaches presented here are unique in that the pulse-echo ambiguity remains and tracking is maintained in spite of it. This allows a much simpler, single frequency system to be more useful.

The biggest limitation of the adaptive delay system is that it can only deal with a small number of aliased echoes. The case when two aliases sandwich the target is dealt with, but if three or more aliases occur in the right spots, there may be no delay time that prevents the target from being obscured.

### Movement

The movement strategy is much different from the other strategies since it cannot be done on a pulse to pulse basis. Moving the sonar to improve sensing also impacts the decisions of navigation that the sensing is intended to facilitate. These results provide more information to consider by the navigation system that must balance sensing and overall task goals. The basic geometry and the angular response of the sonar system suggest that lateral movement with respect to orientation of the sonar is most effective. Another consideration is that any change in sensing angle may, in fact, generate new aliasing problems as it turns to include new background objects. Note that this approach (like the pulse timing method presented in section Adaptive Delay) will have little to no effect for clutter objects that appear at the same range as the target.

### Beam shaping

This technique is a useful way to reduce the effect of aliasing and is the only strategy presented here that is also potentially effective for objects at ranges similar to the target. It is most effective for small angles off-center. Synchronous firing creates a loud central lobe down the central axis of the sonar head. This allows for objects at longer ranges to be detected. This study did not utilize the out of phase firing primarily because the target is assumed to be held in the center of view. The out-of-phase transmission pattern has its minimum in the center of view. If a different tracking algorithm was used that kept the offending clutter in the center, this firing pattern could also be useful in rejecting clutter.

This kind of interference pattern has also been observed to be used by certain bats (Hartley and Suthers, 1987). *Carollia perspicillata* emits sound from two nostril holes. These two nostril holes appear to interact in the same way as depicted in the sonar system above.

### Combining the strategies

While these three strategies have been presented and considered separately, they can be combined into an integrated approach. Adaptive delay and beam shaping can be used simultaneously; the delay can be changed independently of the beam shape. Movements to specifically reduce aliasing can also be made, although other factors will likely affect what actions are taken.

**Figure 15:**
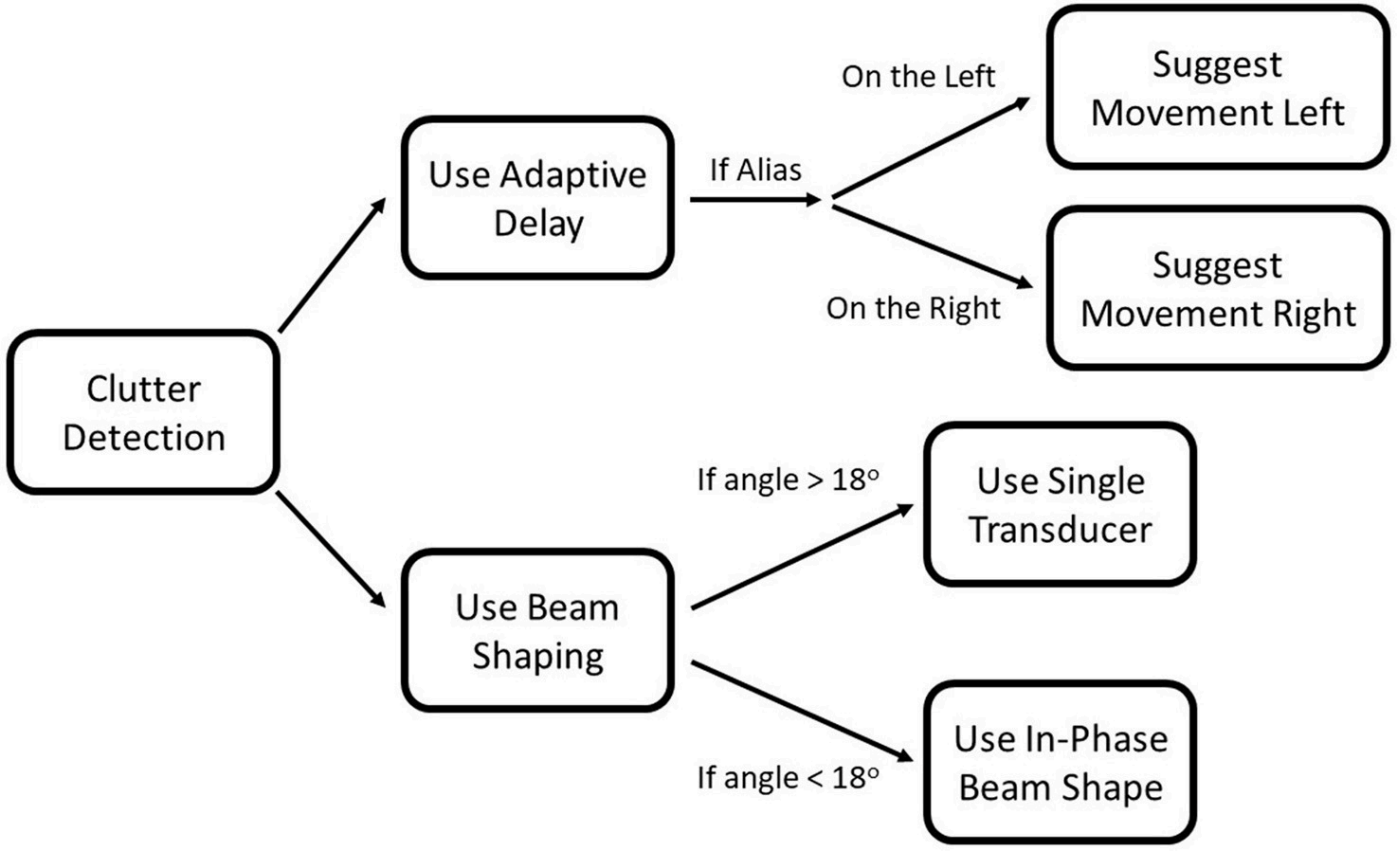
Flowchart for integrating the three strategies. Once clutter is detected, beam shaping, and the adaptive delay can be used simultaneously. If the adaptive delay determines that the object is an alias, a movement direction will be suggested.

If an alias is detected, the adaptive delay approach can be used to prevent the target from being obscured. At the same time, a movement direction can be suggested based on the apparent angle of the alias. If the obstructing echo is determined to be a real object and not an alias (part of the adaptive delay code), then different beam shapes can be used depending on the apparent angle of the obstructing echo. If the angle is less than 18°, synchronous firing will be used. If the angle is greater than 18°, only one transducer will be used. This approach is summarized in Figure 15.

These strategies complement each other well. Together, they present a multi-pronged approach for dealing with the interference produced while using high pulse repetition rates. Each strategy is suited to a different situation and need not be used simultaneously.

## Conclusion

Three different active strategies for dealing with echo aliasing are described that can allow the use of sonar at high sampling rates in cluttered environments. Although a time-domain attentional system is assumed to be able to focus on a specific range to track objects, echoes from clutter objects can overlap in time, obscuring or confusing such an attentional system. At very short interpulse intervals, echoes from the background arriving after the next pulse appear to be at a shorter range then they actually are. These “aliases” can overlap the target and interfere, causing a failure of the tracking system. A dynamic pulse-timing strategy is proposed that can effectively “push” or “pull” the aliased echoes away from the tracked target echo by decreasing or increasing the interpulse interval. This prevents aliases from interfering with tracking. We have also presented a method of avoiding or reducing aliases based on positioning, as well as a method of shaping the echolocation beam to reduce the effect of aliasing or clutter.

Bats have been shown to use several different strategies when encountering cluttered situations that require fast sampling. They have been observed to change the frequency content of consecutive pulses (Hiryu et al., 2010), alternating between short and long pulses (Petrites et al., 2009), and using the directionality of certain harmonics to focus in a given direction (Bates et al., 2011). The system presented here operates on a single carrier frequency, so frequency-based techniques for clutter rejection were not explored, however, we have shown that other techniques are possible (pulse timing, flight steering, and beam shaping) and are possibly also in use by echolocating bats.

## Author contributions

TH: created the hardware and software template for the sonar system; JI: created the sonar mount and modified the software for this project; JI: performed the data collection and analysis with advice and guidance from TH. All authors discussed the results and contributed to the final manuscript.

### Conflict of interest statement

The authors declare that the research was conducted in the absence of any commercial or financial relationships that could be construed as a potential conflict of interest.

## Footnotes

^1^PIC18F2620. (n.d.). Retrieved March 21, 2016, Available online http://www.microchip.com/wwwproducts/en/PIC18F2620.
